# Laparoscopic hysterectomy for benign indications: clinical practice guideline

**DOI:** 10.1007/s00404-017-4467-9

**Published:** 2017-07-26

**Authors:** Evelien M. Sandberg, Wouter J. K. Hehenkamp, Peggy M. Geomini, Petra F. Janssen, Frank Willem Jansen, Andries R. H. Twijnstra

**Affiliations:** 1 Section Minimally Invasive Gynecologic Surgery, Department of Gynecology, Leiden University Medical Center, PO Box 9600, 2300 RC Leiden, The Netherlands; 2 Department of Gynecology, VU University Medical Center, Amsterdam, The Netherlands; 3 Department of Gynecology, Maxima Medical Center, Veldhoven, The Netherlands; 4 Department of Gynecology, Elisabeth-Twee Steden Hospital, Tilburg, The Netherlands; 5 Department of Bio Mechanical Engineering, Delft University of Technology, Delft, The Netherlands

**Keywords:** Laparoscopic hysterectomy, Clinical practice guideline, Best practice, AGREE II tool, GRADE method

## Abstract

**Purpose:**

Since the introduction of minimally invasive gynecologic surgery, the percentage of advanced laparoscopic procedures has greatly increased worldwide. It seems therefore, timely to standardize laparoscopic gynecologic care according to the principles of evidence-based medicine. With this goal in mind—the Dutch Society of Gynecological Endoscopic Surgery initiated in The Netherlands the development of a national guideline for laparoscopic hysterectomy (LH). This present article provides a summary of the main recommendations of the guideline.

**Methods:**

This guideline was developed following the Dutch guideline of medical specialists and in accordance with the AGREE II tool. Clinically important issues were firstly defined and translated into research questions. A literature search per topic was then conducted to identify relevant articles. The quality of the evidence of these articles was rated following the GRADE systematic. An expert panel consisting of 18 selected gynecologists was consulted to formulate best practice recommendations for each topic.

**Results:**

Ten topics were considered in this guideline, including amongst others, the different approaches for hysterectomy, advice regarding tissue extraction, pre-operative medical treatment and prevention of ureter injury. This work resulted in the development of a clinical practical guideline of LH with evidence- and expert-based recommendations. The guideline is currently being implemented in The Netherlands.

**Conclusion:**

A guideline for LH was developed. It gives an overview of best clinical practice recommendations. It serves to standardize care, provides guidance for daily practice and aims to guarantee the quality of LH at an (inter)national level.

## Introduction

Since the introduction of laparoscopic hysterectomy (LH) more than 2 decades ago, a rapid implementation of this procedure has been observed in many countries [1, 2]. For The Netherlands, the percentage of hysterectomies performed laparoscopically has increased from 3% in 2002 to 36% in 2012 [3] and similar increases have been observed in other parts of the world [1, 2]. Such rapid implementation can potentially result in unwarranted practice variations in health care delivery [4]. Unexplained differences in health care delivery should be addressed as they are usually the consequence of a lack of consensus and/or available evidence [5, 6]. Without a convenient standard of care, doctors are more prone to adopt medical practices that are based on personal experience [5, 6]. Furthermore, studies have shown that standardizing care on best practices is associated with better outcomes and reduced costs [7]. As a result, it seems timely to define a standard of care for LH, according to the principles of evidence-based medicine.

With this goal in mind, the Dutch Society of Gynecological Endoscopic Surgery (WGE) initiated the development of a guideline for LH. This guideline aims to provide gynecologists with an overview of best practices, directly applicable for daily practice. The guideline should also ensure a minimum quality of care and enhance patient safety. This article provides a summary of the main recommendations of the guideline.

## Materials and methods

### Development of the guideline

The WGE, a working group of the Dutch Society of Obstetrics and Gynecology (NVOG), initiated the development of the guideline. A guideline working group was assembled and consisted of three gynecologists and one resident (WJKH, PMG, ART and EMS). The guideline was developed in accordance with the Dutch guideline of medical specialists [8]. This document, recognized by all Dutch medical societies, provides a detailed overview of the process of developing an evidence-based guideline using the GRADE method [9]. The Appraisal of Guidelines for Research and Evaluation instrument (AGREE II), an internationally recognized assessment tool, was used in a second stage to evaluate the methodological rigor, transparency and quality of the developed guideline [10]. In the next subsections, the different steps undertaken to create this guideline will be briefly described.

### Step 1: Key topic analysis

A brainstorming session was organized by the WGE with 40 gynecologists, all performing advanced laparoscopic procedures. During that meeting, key topics for this guideline were determined and transformed into appropriate clinical research questions.

### Step 2 and 3: Literature selection, data extraction and assessment of risk of bias

For each research question, a literature search was set up in collaboration with a clinical librarian. PubMed, Medline and Cochrane databases were searched up to 1^st^ of March 2016. Each research question had its own inclusion and exclusion criteria. Overall, we first searched for systematic reviews. If none were available, we focused on randomized controlled trials (RCTs) and, if necessary, added cohort studies as well. Studies from the eligible systematic reviews were reviewed to avoid duplicate inclusions. Only LH for benign indications and/or low-grade malignancy were considered and will hereinafter be referred to as ‘laparoscopic hysterectomy’ (LH). Studies focusing on endometriosis sanitation with concomitant LH as well as high-grade malignancy were not included. Study reports, letters, non-published manuscripts and articles that were not published in English were also excluded. After selecting the eligible studies, these studies were summarized in evidence tables and when possible, extracted for meta-analysis using Review Manager (version 5.2 Copenhagen: The Nordic Cochrane Centre, The Cochrane Collaboration, 2012). The quality of evidence was rated for the different outcomes following the GRADE method [9]. The online GRADE program was used for this purpose (GRADEpro Guideline Development Tool [Software], McMaster University, Hamilton, ON, Canada, 2015, developed by Evidence Prime, Inc., available from gradepro.org).

### Step 4: Concept guideline

From the initial group of 40 gynecologists who participated in the brainstorm session, an expert panel of 18 members was selected. The expert panel and the members of the guideline met a few times to discuss the research questions according to a standard template. The final recommendations were graded according to the classification used by the American Association of Gynecologic Laparoscopists (AAGL) which was adapted from the US Preventive Services Task Force [11]: Level A: Recommendations are based on good and consistent scientific evidence; Level B: Recommendations are based on limited or inconsistent scientific evidence; Level C: Recommendations are based primarily on consensus and expert opinion.

The experts wrote the first draft, after which the working guideline group merged the different topics into one document and finalized the guideline. All experts involved in the development of this document approved the guideline in its present form.

### Step 5: Validation of the guideline

Two independent reviewers, different committees within the NVOG as well as the independent Knowledge Institute of Medical Specialists (KIMS) reviewed the guideline [12]. After approval, our guideline was published on the website of the NVOG to allow all Dutch gynecologists to give feedback. The guideline will be soon adopted in The Netherlands and is valid for 5 years, after which it will be updated. If necessary, it will be (partially) updated earlier.

## Findings

### Overall

For each of the ten main topics raised during the first brainstorm session, a literature search was performed. In total 5233 articles were reviewed and 119 unique articles were included in the guideline. In the following section, each topic and its best practice recommendations are briefly summarized. More detailed information regarding the selected literature, the quality of evidence according to the GRADE method, the search strings of the different topics and the forest plots of the main outcomes, will be published in the fall of 2017 on the website of the NVOG (http://www.nvog.nl).

### Topic 1: A comparison of surgical approaches for hysterectomy

According to the Cochrane review on this topic, vaginal hysterectomy (VH) should be, when technically feasible, the approach of first choice, followed by LH and finally abdominal hysterectomy (AH) [13]. However, limitations of the Cochrane review are the lack of differentiation between the various subtypes of LH (total laparoscopic hysterectomy (TLH); laparoscopic-assisted vaginal hysterectomy (LAVH) and robotic hysterectomy (RH)), and the inclusion of data from older trials performed in the implementation period. Because of these potential limitations, a new literature search was performed for this guideline, specifically comparing TLH to VH. In topic 2, the different subtypes of LH were also compared to TLH. To limit the bias of a learning curve and reflect current practice, we only focused on studies published in the last 15 years (from 1st of January 2000 up to 1st of March 2016).

### TLH versus VH

As can be observed in Table 1, VH was associated with a significantly shorter operative time, a lower risk of conversion and a lower risk of vaginal cuff dehiscence. Patients in the TLH group had lower postoperative pain scores and required analgesia for a shorter period. The other outcomes were similar, and notably the risk of ureter and bladder injury did not differ between the groups, in contrast to what was found in previously published studies [13]. Many factors, such as patient and surgeon characteristics, influence the choice of approach. Our results show that since the implementation of LH, the differences in clinical outcomes between VH and TLH have been minimized. However, when both approaches are feasible, VH is still associated with more relevant benefits compared to LH and should therefore be the approach of first choice.

**Table 1:**
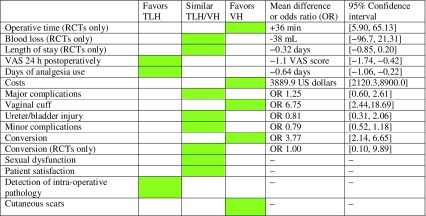
Summary of outcomes comparing TLH to VH

*TLH* total laparoscopic hysterectomy, *VH* vaginal hysterectomy; *RCT* randomized controlled trial

Recommendations



### Topic 2: A comparison of the different subtypes of LH

#### TLH versus LAVH

The percentage of hysterectomies performed using the LAVH technique is decreasing. Currently, LAVHs account for 3% of the LHs in The Netherlands [14]. Based on current literature, no clinically relevant differences were observed between the two approaches. From the meta-analysis performed on this topic, we concluded that the mean differences of 19.7 min (13.08, 26.37) for operative time and 82 ml (−151.95, −12.07) for intra-operative blood loss were not deemed to be clinically relevant. Although the risk of vaginal cuff dehiscence was higher after TLH [OR 2.97 (1.43, 6.18)], the incidence of cuff dehiscence is still low. Furthermore, no overall difference was observed for major complications [OR 1.06 (0.66, 1.68)].

Recommendations



#### TLH versus RH

The results of the meta-analysis showed no clinically relevant differences between TLH and RH for most surgical and patient outcomes. Regarding the costs of the procedure, no meta-analysis could be performed because of incomplete data. Yet, all studies showed that LH was significantly less expensive with mean differences of 1.916 US dollars [15], 3.049 US dollars [16] and 11.214 US dollars [17].

Recommendations



#### TLH versus supra-cervical laparoscopic hysterectomy (SLH)

The results of the meta-analysis for this topic are summarized in Table 2. Despite the fact that most included studies were underpowered and nonrandomized, the expert panel concluded that no major differences were observed between the two procedures, except potentially for complications. In addition, it is important to realize that in the SLH group morcellation is always necessary, which could result in more (mini)laparotomies (topic 8). Finally, the pre-operative cervix cytology, the impact of follow-up screening and the increased risk of cyclic bleeding should also be considered when weighing the pros and cons of the two procedures.

**Table 2:**
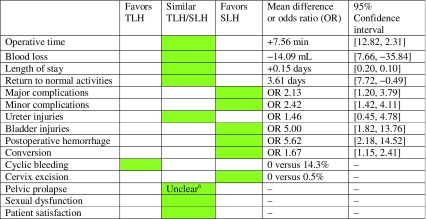
Summary of outcomes comparing TLH to SLH

*TLH* total laparoscopic hysterectomy, *SLH* supra-cervical hysterectomy, *RCT* randomized controlled trial

^a^ Lethaby et al (systematic review): no difference in rate of pelvic prolapse; Berner et al (RCT): higher risk of (asymptomatic) prolapse 12 months after TLH (10% versus 32%)

Recommendations



### Topic 3: What is the added value of pre-operative treatment—gonadotropin-releasing hormone agonists (GnRHa) or ulipristal—prior to LH for uterine fibroids?

This topic evaluated the effect of pre-operative medical treatment (GnRHa and/or Ulipristal) on complication risk, conversion risk, intra-operative blood loss and operative time during LH. The available evidence was limited, especially because many studies did not differentiate between the different approaches of hysterectomy (abdominal, vaginal and laparoscopic). Based on the selected literature, we concluded that there is currently no need to standard pre-operatively treat patients who desire LH for uterine fibroids as the advantages are marginal. However, substantial volume reduction has been demonstrated in some studies (2 weeks in gestational age [18], including a 47% reduction in the study of Donnez et al [19]). Therefore, for each patient a well-considered decision should be made, taking into account the expected volume reduction and hence the possibility for a minimally invasive approach, the side effects and the costs of treatment.

Recommendations



### Topic 4.1: Which patient characteristics influence surgical outcomes during laparoscopic hysterectomy?

To answer this research question, one systematic review was selected [20]. In this review, associations between patient characteristics and surgical outcomes of LH were described based on 85 articles (four RCTs, 29 prospective cohort studies, 47 retrospective cohort studies and five case-control studies).

Recommendations



### Topic 4.2: What is the added value of bimanual examination and medical imaging (ultrasound, MRI) prior to hysterectomy?

Pre-operative gynecological examination (speculum and bimanual examination) gives surgeons information on uterine mobility and an appropriate estimation of the uterine weight. These findings are relevant for determining the operability of the patient (i.e., best surgical approach). Additionally, an ultrasound is useful for detecting potential intra-abdominal pathologies. The expert panel agreed that an MRI is not necessarily superior to ultrasound for hysterectomy with benign indications.

Recommendations



### Topic 5: Which instrument is the most appropriate: bipolar electrothermical energy or ultrasonic energy?

The aim of this topic was to compare bipolar electrothermical energy with ultrasonic energy, particularly with respect to patient safety. Electrothermical energy with monopolar instruments was not included in this topic.

Because of the rapid development of (new) instruments, studies quickly become outdated. The differences observed in surgical outcomes between instruments (bipolar electrothermical energy versus ultrasonic energy) were probably also influenced by surgeon’s experience and preference as well as by the surgical task performed. As differences in clinical findings were small, the expert panel concluded that there was no preference of one instrument over the other. The expert panel emphasized that experience with a specific instrument is valuable and essential for a safe procedure.

Recommendations



### Topic 6: What are the indications for a uterine manipulator and what is its role in preventing ureter injuries?

Recommendations



### Topic 7: Which techniques prevent and/or detect ureter injuries during LH?

#### Ureter stents

As limited evidence was available for benign LH, the search was extended to articles included oncological and endometriosis/DIE cases. Ureter stents do not seem to prevent ureter injury as no significant difference was observed for ureter injuries between the group with and the group without stents [OR 2.45 (0.28; 21.29)]. Standard stent placement could also result in unnecessary complications. Stents are, however, easy to insert and improve the identification of the ureters. In the Delphi study by Janssen et al., the experts did not reach consensus regarding the additional value of ureter stents during LH [21].

Recommendations



#### Cystoscopy

Cystoscopy appears to be safe and results in limited extension of the operative time (mean additional time 13 min). When the overall risk of bladder and/or ureter injuries is below 2%, a standard cystoscopy is not cost-effective for LH [22]. The American Association of Gynecologic Laparoscopists (AAGL) have recommended the standard use of a cystoscopy after LH [23]. The expert panel, on the other hand, concluded that based on available evidence, including incidence data and data on cost-effectiveness, there is insufficient justification to recommend routine cystoscopy after LH. However, the threshold to perform a cystoscopy should be low. When injuries are suspected intra-operatively, additional diagnostics during surgery is recommended and for this a cystoscopy can be of additional value. At last, one should be aware that a normal cystoscopy does not exclude the presence of (lateral thermal) injury, especially for ureter injuries.

Recommendations



#### Intra- and postoperative advice for ureter injuries

Recommendations



### Topic 8: What are the current views regarding power morcellation?

Based on the available evidence, we concluded that the incidence of unexpected sarcoma varies between 1:350 and 1:2000 [24] and increases with age [25]. Other risk factors associated with uterine sarcomas are the following: African race, Tamoxifen use, previous radiotherapy in the pelvic area, HLRCC syndrome and retinoblastoma in the past medical history [25]. The exact impact of malignant spill on overall survival is uncertain, but the risk of upstaging due to morcellation has been estimated to be between 15 and 64% [24]. One of the proposed solutions to minimize spillage of occult malignancy or parasitic myomas is the use of containment bags during morcellation. Although these bags are certainly not optimal yet, they are theoretically able to prevent spread of (malignant) tissue in the abdomen. Gynecologists performing LH should thoroughly counsel their patients and should acquire the skills of in-bag morcellation so that they can offer all the options to their patients. The ESGE developed a flow chart allowing patients to be classified into a low- or high-risk category for sarcomas based on their risk factors and ultrasound results [25]. However, as long as the nature of the uterine mass cannot be diagnosed pre-operatively with certainty, such classifications are not entirely reliable.

Recommendations



### Topic 9: When is the best moment to remove the urinary catheter after LH?

Using a urinary catheter during LH is recommended [26] but the best moment to remove it is unclear. Although evidence was limited, particularly for LH, it seems safe to remove the urinary catheter immediately after hysterectomy. Insufficient evidence was available to determine if leaving the catheter for 6 h offers better outcomes than immediate removal. Leaving the catheter longer than 6 h does not seem to offer any benefits whereas it does increase the risk of urinary tract infection and prolonged hospital stay.

Recommendations



### Topic 10: What advice and/or interventions are helpful to promote postoperative recovery?

Sufficient evidence is available to state that LH is associated with a shorter hospital stay and a quicker recovery than AH [13]. However, research has shown that the time to return to normal activities after LH (i.e., time to return to work) takes overall longer than would be expected [27]. To maximize the benefits of minimally invasive surgery, it is important to adequately guide patients during recovery at home. The complexity of the surgery, the pre-operative expectations of the patient and their pre-operative mental status seem to directly influence the patients’ risk of prolonged absence due to sickness. Therefore, it is important to pre-operatively discuss expectations with the patients. In addition, structured and specific advice results in quicker recovery and E-Health programs can be used for that purpose, Finally, specific advice is needed for each type of hysterectomy as advice is not generalizable for all approaches of hysterectomy [28].

Recommendations



## Discussion and conclusion

This guideline serves as a summary of best practices of LH, and it should provide clinicians with relevant and evidence-based information for daily practice. In other countries such as Germany, guidelines on hysterectomy have been developed as well with similar recommendations [29]. Besides the fact that such guidelines provide surgeons with an overview of the most relevant topics, studies have shown that standardization of care and subsequent guideline compliance is associated with better outcomes and reduced medical liability [30, 31]. Regarding the medico-legal consequences of this guideline, it is probable that in the future it may be used for litigation in The Netherlands. Deviating from this standard of care is obviously allowed, provided that the motivation is thoroughly documented.

Regarding the methodology of this guideline, we focused on systematic reviews and RCTs. If insufficient evidence was available from the RCTs, we added cohort studies to our analysis. A limitation of this approach is that it increases the methodological and clinical heterogeneity. For instance, by including cohort studies, differences in baseline characteristics might exist, which could have influenced the outcomes. On the other hand, this method can also been seen as a strength because for rare events RCTs are often not the best study design as they are often underpowered. During the development of this guideline, we realized that, although GRADE is currently a well-established instrument to assess the quality of evidence [9], it has its limitations as well. The main problem we encountered was that for many topics the available evidence was limited and therefore the quality of the evidence was instantly downgraded to ‘low’ or ‘very low’. This point has been raised previously by other authors [32] and the GRADE working group [33] has stated that on occasion even low available evidence can lead to strong recommendations. The GRADE working group has also emphasized that clinical and cultural settings are of influence and might result in (slightly) different recommendations across countries [33]. Therefore it is essential to choose an expert panel that is well-supported [33]. As the development of our guideline was initiated by the Dutch medical society itself, we believe we had support from the entire country, especially since the panel was a good representation of all Dutch gynecologists.

## Conclusion

The guideline for LH serves as guidance for gynecologists performing LHs. The recommendations in this best practice review should enhance quality of care, minimize (unfavorable) practice variations at the (inter)national level and thereby increase patient safety.
